# Interfacial Behavior During Reactions Between Sn and Electroplated Co–Zn Alloys

**DOI:** 10.3390/ma18122680

**Published:** 2025-06-06

**Authors:** Chao-Hong Wang, Che-Yang Lin

**Affiliations:** Department of Chemical Engineering, National Chung Cheng University, Chiayi 621301, Taiwan; jeff801104@gmail.com

**Keywords:** interfacial reaction, cobalt, electroplating, co-deposition, lead-free solder, suppression

## Abstract

This study investigates the electroplating characteristics of Co-Zn alloy coatings with varying Zn contents (0.55 wt.%~8.8 wt.%) and their influence on intermetallic compound (IMC) formation during reactions with Sn solder. Co-Zn alloy coatings were successfully fabricated by electroplating using cobalt plating solutions with different concentrations of zinc sulfate. The results reveal anomalous co-deposition behavior, where the less noble Zn preferentially deposits over Co. Surface morphologies and microstructures evolve significantly with increasing Zn content, transitioning from columnar to dendritic structures. Zn incorporation into the Co lattice disrupts its crystallinity, leading to decreased crystallinity and partial amorphization. Liquid-state and solid-state interfacial reactions with Sn solder demonstrate that Zn content considerably influences IMC formation. In liquid-state reactions at 250 °C, lower Zn contents (0.55–4.8 wt.%) slightly enhance CoSn_3_ growth. It exhibits a dense layered-structure without IMC spallation. In contrast, a higher Zn content (8.8 wt.%) significantly reduces IMC formation by approximately 50% and produces a duplex structure with two distinct layers. In solid-state reactions at 160 °C, the suppression effect becomes even more pronounced. The Co-0.55Zn deposit exhibits significant inhibition of CoSn_3_ growth, while the Co-8.8Zn sample forms only a thin IMC layer, achieving a suppression rate exceeding 85%. These findings demonstrate that Zn doping effectively limits CoSn_3_ formation during solid-state reactions and improves interfacial stability.

## 1. Introduction

Soldering technology plays a crucial role in microelectronic packaging, driving the advancement of various packaging technologies. Numerous tiny solder balls form solder joints that interconnect integrated circuit (IC) chips with IC substrates, such as in flip-chip packaging, or with printed circuit boards (PCBs) in ball grid array (BGA) technology [1,2,3]. As electronic devices continue to shrink in size, solder joints are being progressively miniaturized, raising increasing concerns about their reliability. To prevent the rapid dissolution of Cu pads by lead-free solders during reflow soldering, Ni is commonly used as a diffusion barrier. Among the various surface finishes developed for Cu pads, the electroless nickel immersion gold (ENIG) is one of the most widely adopted solutions [4,5,6]. Extensive studies have been conducted on the interfacial issues between lead-free solders and Ni or other related surface finish substrates [7,8,9,10,11,12].

In addition to Ni, Co has emerged as a promising alternative diffusion barrier due to its excellent electromigration resistance and the superior mechanical properties of Sn-Co IMCs, which are beneficial for the reliability of solder joints [13,14,15,16]. Similar to electroless Ni(P), electroless Co(P) deposition has been developed for practical applications on solder pads [17,18,19,20]. The interfacial reactions between lead-free solders and Co or its related alloys have also been investigated [21,22,23,24,25], offering valuable insights for assessing their practical applicability. Nevertheless, Co exhibits faster IMC growth with solder compared to Ni [24,25]. Furthermore, in electroless Co(P) deposits, substantial IMC spalling has been observed during soldering, leading to rapid consumption of the Co(P) layer. This degradation can severely compromise the interfacial reliability of the solder joints [18,22,26]. As a result, ongoing research efforts are focused on addressing IMC spalling and suppressing IMC growth to improve the performance of Co-based diffusion barriers in soldering applications.

The minor addition of elements, such as Zn and Cu, into solder has been found to effectively suppress CoSn_3_ growth at the solder/Co interface during soldering [16,27,28]. For instance, adding 0.5 wt.%Zn to Sn-based solder at 250 °C resulted in a significant reduction of approximately 75% in CoSn_3_ growth [27]. This observation suggests that Zn doping in Co-Zn alloys may also affect the interfacial behavior of Sn-Co IMCs. This indicates that Zn may play a critical role in modifying interfacial IMC behavior. Inspired by this, Zn doping directly into the Co substrate may also influence IMC formation, although this has not been thoroughly investigated.

This study aims to investigate the interfacial reactions between Sn-based solder and Co-Zn alloy coatings with varying Zn contents. The Co-Zn coatings were fabricated via electroplating in a cobalt sulfamate-based bath containing different concentrations of zinc sulfate, with the Zn content controlled to be below 10 wt.%. Due to the known anomalous co-deposition behavior in Co-Zn systems [29,30,31], the less noble Zn may preferentially deposit over the more noble Co, resulting in a higher Zn content in the deposit than predicted. While the prior study has focused on the addition of Zn into solder, the effects of Zn incorporated directly into the substrate (i.e., the metallization layer) on interfacial IMC formation remain unclear. Therefore, a systematic investigation was conducted to examine how varying Zn concentrations in the Co-Zn coatings influence the growth behavior of intermetallic compounds. This work reveals that increasing Zn content in the Co-Zn coatings significantly suppresses CoSn_3_ growth during soldering. A nucleation-based mechanism is proposed to explain the suppression behavior.

## 2. Materials and Methods

To fabricate Co-Zn alloy coatings onto Cu substrates, zinc sulfate (ZnSO_4_·7H_2_O) was added to a basic Co electroplating bath based on a cobalt sulfamate (Co(SO_3_NH_2_)_2_) system. The constituents of the Co electroplating solution are listed in Table 1. Four Co-Zn alloy coatings were prepared by introducing different amounts of ZnSO_4_·7H_2_O, 0.1 g, 0.5 g, 1 g, and 2 g, corresponding to Zn^2+^ concentrations of 0.0017 M, 0.0087 M, 0.017 M, and 0.035 M, respectively, into 200 mL of the plating solution, as summarized in Table 2. Each plating solution was maintained in a 55 °C water bath and thoroughly stirred to ensure complete dissolution of ZnSO_4_·7H_2_O. Before electroplating, a 0.5 mm-thick Cu substrate was cut into 10 mm × 15 mm pieces. Each specimen was mechanically polished with alumina powder and then ultrasonically cleaned in deionized (DI) water. The plating window was controlled to a size of 7 mm × 10 mm. During the Co-Zn electrodeposition process, the Cu substrate and a Co plate served as the cathode and anode, respectively, positioned 6 cm apart. Electroplating was carried out at 55 °C under a current density of 2 ASD (ampere per square decimeter) for 25 min. The theoretical deposited thickness was ~10 µm.

The top-view morphology of the Co-Zn deposits was examined using scanning electron microscopy (SEM), and their composition was analyzed with an electron probe microanalyzer (EPMA, JEOL JXA-8200, JEOL Ltd., Tokyo, Japan). The deposits were characterized by X-ray diffraction (XRD, Bruker D8) analysis with Cu-K_α_ radiation (λ = 0.154056 nm). The diffraction patterns were identified by comparison with reference patterns from the Joint Committee on Powder Diffraction Standards (JCPDS) database. For cross-sectional observation of the grain structure, the Co-Zn deposits were examined using focused ion beam (FIB) microscopy (FEI Quanta 3D FEG, Thermo Fisher Scientific, Waltham, MA, USA). The samples were lightly polished to obtain a flat surface. A protective Pt layer was then deposited, followed by FIB milling. The grain structure of the Co-Zn coatings was observed using ion-induced secondary electron (ISE) imaging.

For the soldering reaction, the Co-Zn deposits were polished to obtain a flat surface and then reacted with Sn at 250 °C for durations ranging from 10 s to 2 h. In addition to the soldering reaction, solid-state reactions were also investigated. To prepare the reaction couples, Sn solder was reflowed onto the Co-Zn deposits at 250 °C for 3 s to form an initial joint. Subsequently, the samples were aged at 160 °C on a hot plate for predetermined durations, such as 160 h. After the reaction, the samples were mounted in epoxy resin, and then ground and polished for metallographic analysis. The polished samples were lightly etched with an Sn etchant to enhance the visibility of the interfacial zone. The interfacial morphologies were then examined using back-scattered electron imaging (BEI) mode in SEM. The composition of the reaction phases was analyzed using EPMA. The thickness of the reaction phase was determined by calculating the total area of the phase using image analysis software (Optimas version 6.1, Optimas Corporation, Wood Dale, IL, USA) and dividing it by the measured interface length.

## 3. Results and Discussion

### 3.1. Characterization of Electroplated Co-Zn Coatings

**Figure 1:**
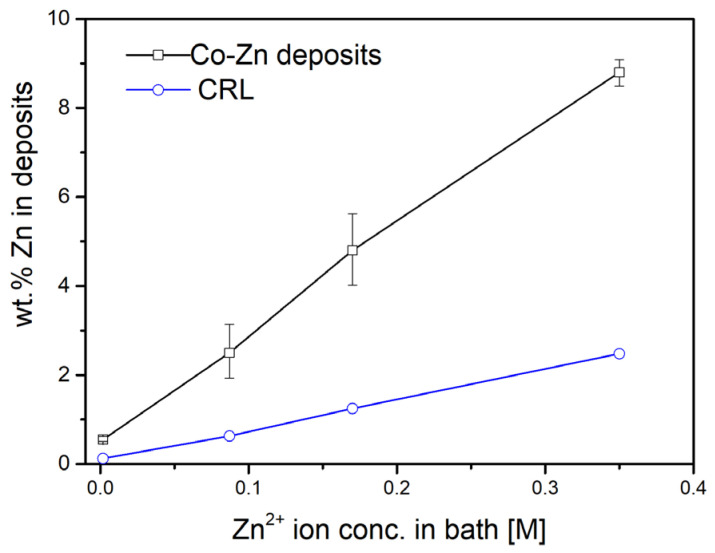
Zn concentrations in the Co-Zn deposits as a function of Zn^2+^ ion concentration in the electroplating bath.

As described in the experimental methods, four Co-Zn alloy coatings with varying Zn contents were successfully fabricated by electroplating, in which different amounts of zinc sulfate were added to 200 mL of a Co electroplating solution. The chemical compositions of the resulting coatings, determined by EPMA measurements at multiple locations across the cross-sections, are summarized in Table 2. The coatings were denoted as Co-0.55 wt.%Zn, Co-2.5 wt.%Zn, Co-4.8 wt.%Zn, and Co-8.8 wt.%Zn, respectively. As shown in Figure 1, the Zn content in the deposits increases nearly linearly with increasing Zn^2+^ concentration in the plating bath, corresponding to the amount of zinc sulfate added. The relatively narrow compositional ranges suggest that the Zn distribution within each coating is reasonably uniform, with no apparent segregation or compositional inhomogeneity observed.

In Figure 1, the compositional reference line (CRL) represents the predicted Zn content in the deposits, calculated based on the concentration ratio of Zn^2+^ ions to the total metal ion concentration (Co^2+^ and Zn^2+^) in the plating solution. Notably, the experimentally measured Zn contents were consistently higher than the CRL, suggesting that Zn, being electrochemically less noble than Co, was preferentially deposited during the electroplating process. This behavior was characteristic of anomalous co-deposition [29,30]. The Zn contents in the deposits were approximately 4.4, 3.8, 3.8, and 3.5 times higher than the CRL values, respectively, showing a slight downward trend with increasing Zn^2+^ concentration in the plating bath.

The observed anomalous co-deposition behavior, where Zn exhibits a greater tendency to deposit than the more noble Co, can be attributed to several electrochemical factors, including localized pH increases and kinetic effects. Although Zn has a more negative standard reduction potential (−0.76 V for Zn^2+^/Zn vs. −0.28 V for Co^2+^/Co), it can still be preferentially deposited due to local pH changes near the cathode surface. The increase in local pH promotes the precipitation of Zn(OH)_2_, which accumulates on the cathode surface and acts as a barrier, hindering the discharge of Co^2+^ ions and thus suppressing Co deposition [32,33,34]. Additionally, Zn exhibits faster deposition kinetics than Co, which further enhances its preferential deposition during the co-electroplating process [29].

**Figure 2:**
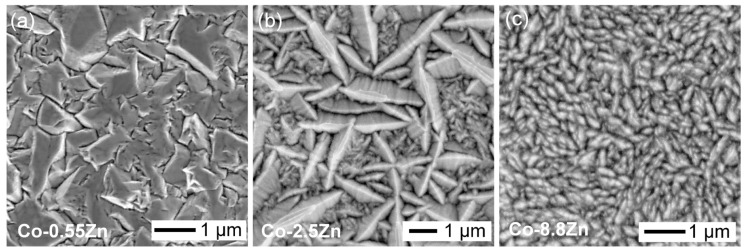
Top-view SEM images of the electroplated Co-Zn surfaces with different Zn contents: (**a**) Co-0.55Zn, (**b**) Co-2.5Zn, and (**c**) Co-8.8Zn.

Figure 2a–c shows the surface morphologies of electroplated Co-Zn deposits with Zn contents of 0.55 wt.%, 2.5 wt.%, and 8.8 wt.%, respectively. Significant changes in surface morphology were observed with increasing Zn content. A rough and irregular surface texture was observed in the Co-0.55Zn deposit, composed of large, angular, faceted grains approximately 1 µm in size with well-defined boundaries. In contrast, the Co-2.5Zn deposit displayed densely packed, elongated, needle-like grains, that formed a more interconnected and textured structure. Notably, two distinct grain size populations were observed: larger grains (~2 µm) and finer grains, with sizes of several hundred nanometers. As the Zn content increased, the grains became progressively finer, and the fraction of larger grains decreased. In the Co-8.8 wt.%Zn coating, the surface consisted of uniformly distributed fine grains, leading to a smoother and more homogeneous morphology.

**Figure 3:**
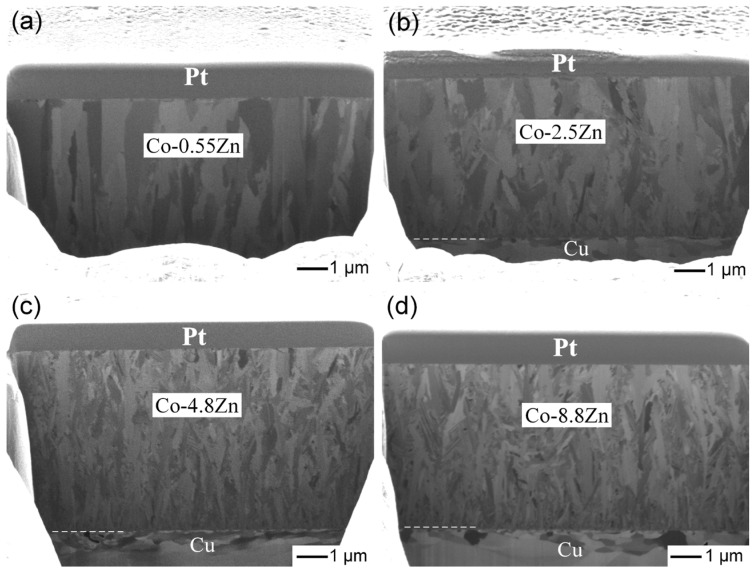
FIB cross-sectional images of the electroplated Co-Zn deposits: (**a**) Co-0.55Zn, (**b**) Co-2.5Zn, (**c**) Co-4.8Zn, and (**d**) Co-8.8Zn.

Cross-sectional observations of the coatings were performed using FIB to investigate the structural evolution of Co-Zn coatings with varying Zn contents, as shown in Figure 3a–d. At low Zn content (0.55 wt.%), the coating exhibited coarse and straight columnar grains. As the Zn content increased to 2.5 wt.% and 4.8 wt.%, the columnar structure was maintained, but the grains became more disordered and refined. At the highest Zn content (8.8 wt.%), the columnar grains evolved into a finer dendritic morphology, suggesting that Zn incorporation promoted grain refinement and disrupted the typical columnar growth pattern. These microstructural changes were consistent with the variations observed in the surface morphologies as the Zn content increased.

**Figure 4:**
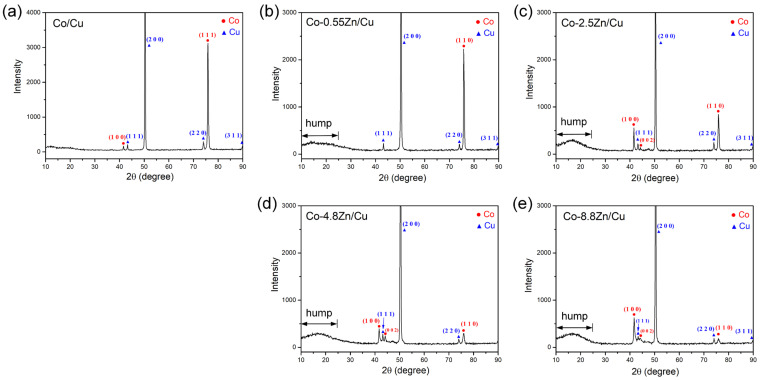
XRD patterns of the electroplated Co-Zn deposits on Cu substrate, (**a**) Co, (**b**) Co-0.55Zn, (**c**) Co-2.5Zn, (**d**) Co-4.8Zn, and (**e**) Co-8.8Zn.

To further assess the crystallinity and crystallographic orientation of the Co-Zn deposits, XRD analyses were conducted, with a pure Co deposit used as a reference, as shown in Figure 4a–e. In the XRD pattern of the Co-0.55Zn deposit, the hcp-Co peaks (JCPDS #89-4308) were significantly weaker than those of the underlying fcc-Cu substrate (JCPDS #89-2838), indicating a substantial reduction in the crystallinity of the Co phase. Notably, a distinct broad hump was observed in the 2θ range of 10° to 25°, which was characteristic of an amorphous structure. This feature was absent in the pure Co deposit and was thus attributed to the incorporation of Zn atoms into the hcp-Co lattice, which disrupted long-range atomic ordering. Although both Zn and Co possess hcp crystal structures, the larger atomic radius of Zn induced severe lattice distortion, leading to a loss of crystallinity. Despite this reduction, a preferred orientation along the (110) plane of hcp-Co at a 2θ value of 75.8° remained observable, similar to that in the pure Co deposit.

As the Zn content increased to 2.5 wt.%, the intensity of the Co peaks further decreased, and the broad hump became more pronounced. This indicated that Zn incorporation into the Co lattice further reduced the crystallinity of the Co phase and induced partial amorphization. In addition to the (110) peak of hcp-Co, a prominent (100) peak appeared at 2θ = 41.6°. Similar features were observed in the XRD patterns of the Co-4.8Zn and Co-8.8Zn deposits. In contrast, in the XRD spectra, the (002) peak remained much weaker, suggesting that the Co-Zn coatings preferentially grew along the (100) and (110) planes, with the c-axis of the hcp-Co structure oriented parallel to the substrate. The XRD analysis revealed a noticeable reduction in crystallinity with increasing Zn content in the Co-Zn coatings. This structural change suggests the potential for enhanced hardness and strength, as amorphous or nanocrystalline structures typically hinder dislocation motion. However, it should be noted that such microstructural characteristics may also result in increased internal stresses and reduced toughness, which could negatively affect the mechanical reliability of the coatings.

### 3.2. Interfacial Reactions of Co-Zn Deposits with Sn at 250 °C

**Figure 5:**
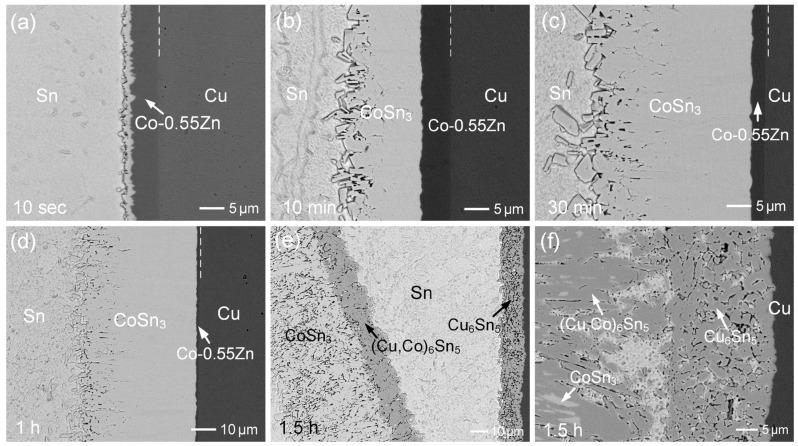
BEI micrographs of the Sn/Co-0.55Zn reaction at 250 °C for different durations: (**a**) 10 s, (**b**) 10 min, (**c**) 30 min, (**d**) 1 h, (**e**) 1.5 h, and (**f**) magnified view of the sample after 1.5 h.

Figure 5a–f shows the interfacial microstructures of the Sn/Co-0.55Zn reactions at 250 °C for various durations, ranging from 10 s to 1.5 h. After just 10 s, a thin reaction layer was formed. Notably, the interface between the IMC and the Co-0.55Zn coating appeared irregular, suggesting slight dissolution of the Co-0.55Zn into the solder during the initial reaction stage. After 10 min of aging, a bright and significantly thicker IMC layer, approximately 15 µm, was observed. EPMA analysis indicated that the IMC consisted of Sn-25.06 at.%Co and 0 at.%Zn, and was identified as the CoSn_3_ phase, with no detectable Zn content. The IMC grew rapidly; by 30 min (Figure 5c), the layer reached ~31 µm in thickness. With further aging to 1 h (Figure 5d), the dense reaction phase thickened further to approximately 45 µm.

**Figure 6:**
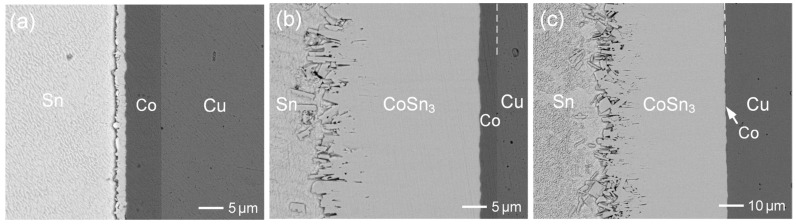
BEI micrographs of the Sn/Co reaction at 250 °C for (**a**) 10 s, (**b**) 1 h, and (**c**) 2 h.

For comparison, the reaction was also performed using electroplated pure Co, as shown in Figure 6a–c. As seen in Figure 6b, the CoSn_3_ layer reached a thickness of approximately 34 µm. The IMC layers formed after 10 min and 30 min of aging were relatively thinner than those observed in the Co-0.55Zn reaction. These results suggested that the addition of 0.55 wt.%Zn enhanced the reaction kinetics and promoted IMC growth. In Figure 6a, the interface between the IMC and pure Co appeared relatively straight, further supporting the earlier suggestion that the Zn addition accelerated the dissolution of the Co-Zn alloy into molten Sn. After 2 h of aging (Figure 6c), the IMC layer became very thick (~55 µm), although a thin layer of the Co deposit still remained.

For the Sn/Co-0.55Zn reaction, as the reaction time was extended to 1.5 h, the Co-0.55Zn layer was completely depleted, and the formed CoSn_3_ layer detached from the Cu substrate, as shown in Figure 5e. EPMA analysis revealed that the bright phase of the detached layer had a composition of Sn-24.3 at.%Co-1.0 at.%Cu-0.1 at.%Zn, corresponding to the CoSn_3_ phase. The adjacent dark phase exhibited a composition of Sn-10.7 at.%Co-42.7 at.%Cu-0.02 at.% Zn and was identified as the (Cu,Co)_6_Sn_5_ phase. On the Cu substrate side, the dark phase consisted of numerous Cu_6_Sn_5_ grains with a composition of Sn-2.1 at.%Co-46.7 at.%Cu-0.13 at.%Zn. Since the Co content in these grains was relatively low, they were designated as Cu_6_Sn_5_ to distinguish them from the dispersed (Cu,Co)_6_Sn_5_ phase with higher Co solubility. Figure 5f shows a magnified view of the (Cu,Co)_6_Sn_5_ phase and the porous Cu_6_Sn_5_ phase. The (Cu,Co)_6_Sn_5_ phase exhibited a striped morphology, with the CoSn_3_ phase surrounded by (Cu,Co)_6_Sn_5_. This observation suggested that the formation of the (Cu,Co)_6_Sn_5_ phase likely occurred via a transformation of CoSn_3_, induced by Cu dissolution from the substrate. Additionally, it is noteworthy that the porous Cu phase maintained a stable layered morphology. The grains remained intact and did not disintegrate or spall into the solder.

**Figure 7:**
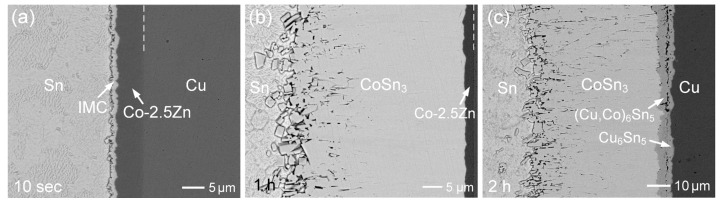
BEI micrographs of the Sn/Co-2.5Zn reaction at 250 °C for (**a**) 10 s, (**b**) 1 h, and (**c**) 2 h.

With an increase in Zn content to 2.5 wt.%, the interfacial microstructures were examined. As shown in Figure 7a, the initial interfacial structure resembled that of the Co-0.55Zn reaction. The Co-2.5Zn deposit was unevenly consumed due to dissolution, resulting in a rough and irregular interface. As the reaction progressed, the CoSn_3_ layer thickness increased to approximately 13 µm after 10 min of aging and to around 32 µm after 30 min (Figure S1). The CoSn_3_ phase contained a minor amount of 0.26 at.%Zn. As shown in Figure 7b, the thickness of the CoSn_3_ layer after 1 h of aging was comparable to that observed in the Co-0.55Zn reaction.

**Figure 8:**
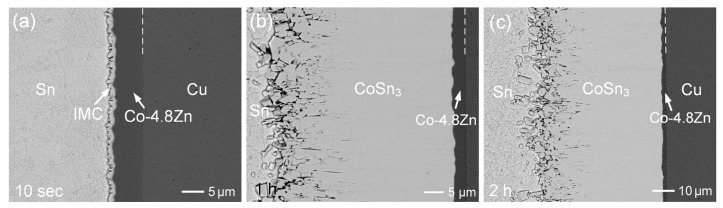
BEI micrographs of the Sn/Co-4.8Zn reaction at 250 °C for (**a**) 10 s, (**b**) 1 h, and (**c**) 2 h.

After reacting for 2 h (Figure 7c), the deposited layer was completely consumed, and the Cu substrate began to participate in the reaction. EPMA analysis, as shown in Table S1, indicated that the resulting reaction phases were CoSn_3_, (Cu,Co)_6_Sn_5_, and Cu_6_Sn_5_. A crack was observed at the interface between the (Cu,Co)_6_Sn_5_ and Cu_6_Sn_5_ phases, leading to the detachment of the reaction layer from the substrate. Furthermore, as shown in Figure 8 and Figure S2, the Co-4.8Zn deposit also exhibited similar interfacial behavior and a comparable trend in IMC growth rate to that observed in the Co-2.5Zn reaction. In all three Co-Zn coatings, Sn atoms from the solder diffused through the CoSn_3_ layer and subsequently reacted with the Co-Zn substrate to form a new CoSn_3_ phase.

**Figure 9:**
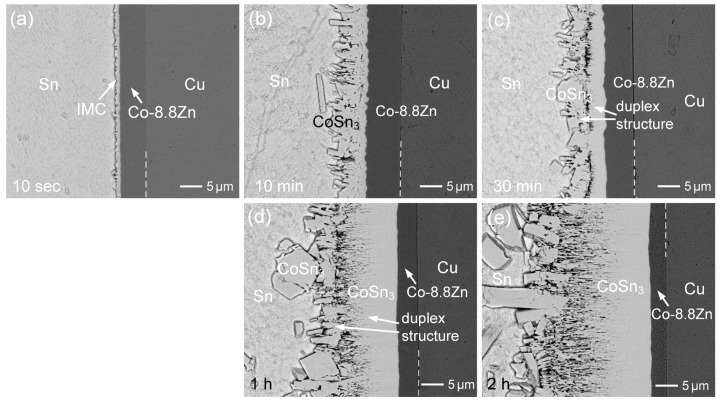
BEI micrographs of the Sn/Co-8.8Zn reaction at 250 °C for (**a**) 10 s, (**b**) 10 min, (**c**) 30 min, (**d**) 1 h, and (**e**) 2 h.

For the Zn content of 8.8 wt.%, the interfacial morphologies at various reaction durations are shown in Figure 9a–e. After aging for 10 min and 30 min, the IMC thickness increased to approximately 6 µm and 7.5 µm, respectively. These values were significantly lower compared to the IMC growth observed in the other three Co-Zn coatings. This suggests that the 8.8 wt.%Zn content was effective in suppressing IMC growth by ~50%, compared to the Sn/Co reaction. The prior study [27] reported that even a low Zn concentration of 0.1 wt.% in Sn solder could significantly inhibit CoSn_3_ growth by 43%, and that increasing the Zn content to 0.5 wt.% further enhanced the suppression effect to 76%. The minor Zn addition in solder was suggested to inhibit the chemical reaction responsible for the nucleation and formation of CoSn_3_. Consequently, the dissolution of the Co-8.8Zn deposit into the solder likely increased the Zn concentration, the dissolution of the Co-8.8Zn deposit into the solder likely increased the Zn concentration, leading to further suppression of IMC growth. Additionally, EPMA analysis in Figure 9b revealed that the CoSn_3_ phase contained approximately 0.45 at.%Zn, which aligns with the findings from the prior study [27]. Although the Zn concentration in the CoSn_3_ phase was slightly higher than that observed in the other three Co-Zn coatings, it remained at a very low level, suggesting that most of the Zn from the Co-Zn deposits dissolved into the solder.

To better understand the influence of Zn dissolution from the Co-Zn coating into the solder, a simplified estimation was performed. Assuming that a 1 μm thick Co-8.8 wt.%Zn layer dissolves into a 100 μm thick interfacial region of molten Sn, the resulting average Zn concentration in the solder is calculated to be approximately 0.107 wt.%. Although this concentration is relatively low, it may still affect the nucleation and growth behavior of IMC at the interface. This estimation is consistent with previous findings [27], where even small additions of Zn (~0.1 wt.%) have been reported to suppress IMC growth. Furthermore, the suppression of excessive CoSn_3_ growth in Co-Zn coatings can be primarily attributed to the influence of Zn on the nucleation behavior of CoSn_3_. During the interfacial reaction, Sn acts as the dominant diffusing species, and the growth front of CoSn_3_ is located at the interface between the CoSn_3_ layer and the underlying Co-Zn coating. Zn atoms, either incorporated within the coating or segregated at the interface, can alter the interfacial energy and local chemical environment, increasing the nucleation energy barrier for CoSn_3_ formation. As a result, the number of active nucleation sites is reduced and the initial formation rate of CoSn_3_ is slowed, which further suppresses the overall IMC growth kinetics.

As presented in Figure 9b,c, the IMC phase displayed a distinct duplex morphology, with two distinct microstructures separated by a crack. This implies that different formation mechanism governed the development of the IMC phase. The inner CoSn_3_ layer, formed adjacent to the Co-8.8 wt.%Zn deposit, was formed through the diffusion of Sn and its subsequent reaction with the Co-Zn coating at the interface. However, its growth was notably suppressed, especially during the initial reaction stage. On the other side, the IMC phase near the solder exhibited large grains, likely formed through the dissolution of the underlying CoSn_3_ layer, followed by reprecipitation and grain growth. The crack separating the inner and outer CoSn_3_ layers is likely induced by growth stress generated during the formation of the inner layer at the interface between the Co-Zn coating and the IMC. The volumetric expansion associated with CoSn_3_ formation, combined with constrained growth conditions, can lead to the accumulation of internal stress. When this stress exceeds the local mechanical strength, it may result in interfacial cracking or delamination between the two IMC sublayers. After prolonged reaction times of 1 h and 2 h, as shown in Figure 9d,e, respectively, the microstructure remained unchanged. However, the CoSn_3_ phase on the solder side exhibited significantly larger faceted grains, while the inner CoSn_3_ layer, which displayed a striped grain structure, showed substantial growth. In the later stage of the reaction, the suppression effect significantly decreased, and the growth of the inner CoSn_3_ phase became dominant.

The Co-Zn coatings demonstrated good adhesion to the Cu substrate across all Zn contents during the experimental process. Even after high-temperature interfacial reactions, quenching, and subsequent metallographic preparation, no obvious delamination was observed. Typically, higher doping levels (e.g., increased Zn content), elevated plating current densities, and thicker deposits can induce greater internal stress within the coating, which may compromise adhesion and lead to peeling. Therefore, special attention must be paid to controlling internal stress during the coating fabrication process. In Figure 9c, a seam resembling peeling was observed at the interface between the Co-8.8Zn deposit and the Cu substrate. However, this feature is considered a superficial surface artifact rather than a true interfacial separation. A more detailed discussion is provided in Figure S3 of the Supplementary File.

The thermal expansion mismatch was further analyzed. Table 3 summarizes the coefficients of thermal expansion (CTE) of the relevant materials. The mismatch between the Cu substrate and the Co-Zn coatings is relatively minor and unlikely to induce significant thermal stress. In contrast, a more substantial mismatch exists between the Sn solder and the Co-Zn/Cu substrate, which may generate considerable thermal stress during cooling from the soldering temperature. Notably, the CTE of Co-Zn alloys is lower than that of Sn and closer to that of Cu, potentially helping to mitigate thermal strain at the solder/coating interface. Despite this inherent thermal mismatch, no evident delamination was observed, indicating that the Co-Zn coatings maintained good mechanical integrity throughout the soldering process.

**Figure 10:**
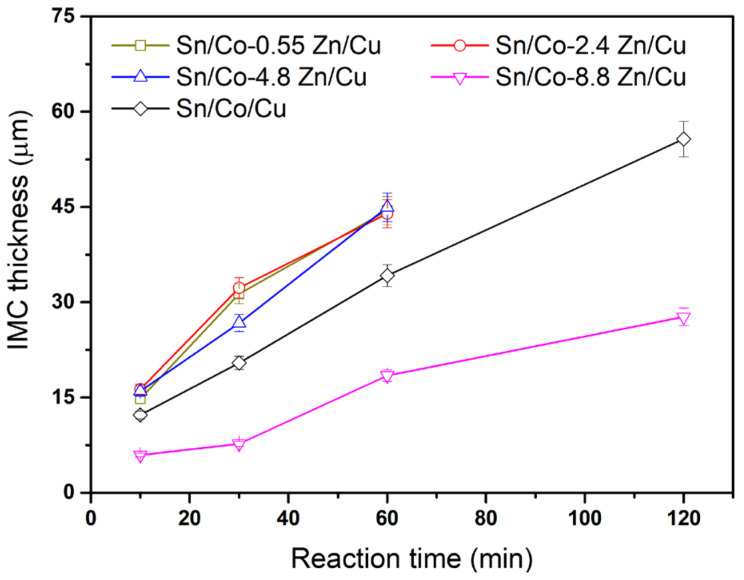
IMC thickness as a function of reaction time in the reactions between Sn and Co-Zn deposits.

Figure 10 shows the IMC thickness as a function of reaction time for various Co-Zn deposits. For the Co-0.55Zn, Co-2.5Zn, and Co-4.8Zn samples, the IMC growth rate was slightly higher than that observed for pure Co. This unexpected trend could be attributed to the insufficient Zn content in these alloys, which was likely inadequate to form a continuous or effective barrier at the interface. At low Zn concentrations (e.g., 0.55–4.8 wt.%), Zn likely promotes the initial dissolution of the Co-Zn deposits into the molten Sn, thereby increasing the local availability of Co atoms and accelerating the formation of the CoSn_3_ phase. In this regime, the Zn content is insufficient to significantly alter the interfacial chemistry or form a continuous Zn-rich barrier layer. As a result, the dominant growth mechanism remains similar to that of pure Co, and the slight enhancement in IMC thickness can be attributed to the more efficient supply of Co into the solder matrix. Conversely, the Co-8.8Zn alloy exhibited significantly suppressed IMC growth. This may result from the higher Zn content altering the interfacial chemistry, which hinders the nucleation of CoSn_3_. Moreover, high Zn content may have promoted the dissolution and subsequent reprecipitation of CoSn_3_ into coarser grains, leading to a slower net growth rate of the IMC layer. These results indicate that only when the Zn content exceeds a certain threshold (e.g., 8.8 wt.%) does Zn begin to exert a pronounced suppression effect.

In addition to Zn, other alloying strategies have also been investigated for suppressing interfacial IMC growth. Electroless Co-P coatings, typically deposited using sodium hypophosphite as a reducing agent, usually contain high phosphorus contents (~6 wt.%), which have been reported to accelerate interfacial reactions rather than suppress them. In contrast, electroplated Co-P coatings with a low phosphorus content (e.g., 0.7 wt.%) have been shown to effectively suppress IMC growth [18]. Notably, the effect of P doping exhibits an opposite trend compared to that of Zn in the Co-Zn system, where a higher Zn content leads to a stronger suppression effect.

### 3.3. Interfacial Reactions of Co-Zn Deposits with Sn at 160 °C

**Figure 11:**
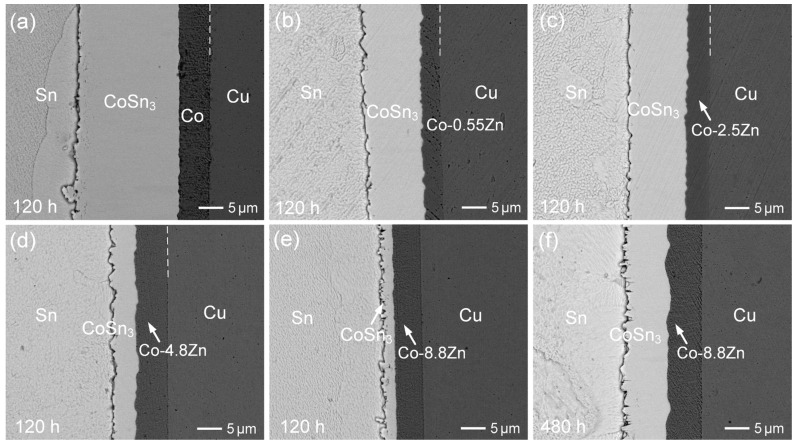
BEI micrographs of the interfacial reactions between Sn and various Co-Zn deposits after aging at 160 °C for 120 h: (**a**) Co, (**b**) Co-0.55Zn, (**c**) Co-2.5Zn, (**d**) Co-4.8Zn, and (**e**) Co-8.8Zn. (**f**) Co-8.8Zn after 480 h.

The solid-state interfacial reactions between Sn and various Co-Zn deposits were further investigated at 160 °C. Figure 11a–e present the interfacial microstructures after aging for 120 h. Both the Co-0.55Zn and Co-2.5Zn samples exhibited similar CoSn_3_ layer thicknesses of approximately 11 µm, which were significantly thinner than that of the pure Co sample (~20 µm). This indicates that, in the solid-state reaction, even small amounts of Zn in the Co deposit were effective in hindering the formation of IMCs. Moreover, the suppression effect became more pronounced with increasing Zn content. The Co-4.8Zn sample exhibited an even thinner IMC layer, measuring only ~5 µm, representing a reduction of approximately 75% compared to pure Co. This suggests that Zn likely affects the nucleation or growth rate of CoSn_3_ and possibly alters the diffusion kinetics or the interaction of Sn with the Co-Zn deposits.

Figure 11e further confirms this trend, showing that the Co-8.8Zn sample exhibited a significantly reduced IMC thickness of approximately 2.5 µm after aging for 120 h. Even after extended aging for 480 h, the IMC layer grew to only around 9 µm, indicating that the suppression remained effective. These results clearly demonstrate that the inhibition of IMC growth in the solid-state reaction is considerably more pronounced than in the liquid-state reaction. This may be attributed to the slower diffusion rates at lower temperatures, which provide more time for Zn to affect the interfacial reaction. Additionally, these findings suggest that optimizing the Zn content in Co-Zn alloys could be a promising strategy for controlling IMC formation in soldering processes, especially for applications that require long-term reliability.

This study primarily focuses on the fabrication of Co-Zn deposits and the interfacial reaction behavior of Co-Zn coatings during the liquid-state reflow process. Compared to conventional Ni, Co, and Co-P systems, the Co-Zn alloy exhibits unique alloying effects and enhanced interfacial stability. The Co-Zn system not only maintains excellent barrier properties and thermal stability but also offers the potential to modulate interfacial reaction kinetics through Zn addition, effectively suppressing the formation of detrimental phases. Furthermore, Co-Zn alloys demonstrate promising diffusion barrier performance and solder compatibility, which can contribute to improved reliability in electronic packaging interconnects.

To fully evaluate the practical applicability of these coatings, future work will include long-term aging experiments and mechanical reliability assessments of solder joints with varying Zn contents. Such investigations are essential for verifying the durability and performance of Co-Zn coatings under practical service conditions, thereby further supporting their potential for industrial applications in microelectronic packaging. Moreover, further studies are necessary to elucidate the fundamental mechanisms by which Zn influences interfacial reactions, particularly its effects on diffusion behavior and phase formation.

## 4. Conclusions

This study successfully fabricated Co-Zn alloy coatings with varying Zn contents via the electroplating method. The compositional trends of the coatings exhibited an anomalous co-deposition behavior, where the less noble Zn had a greater tendency to deposit than Co. Increasing Zn content significantly altered the surface morphologies and cross-sectional microstructures, resulting in finer grains and a transition from columnar to dendritic structures. XRD analysis revealed that Zn incorporation reduced the crystallinity of the Co phase and induced partial amorphization. Interfacial reaction studies with Sn solder at 250 °C and 160 °C demonstrated the critical role of Zn in IMC formation. At 250 °C, lower Zn contents (0.55–4.8 wt.%) slightly accelerated CoSn_3_ growth due to enhanced dissolution of the Co-Zn layer. However, higher Zn content (8.8 wt.%) significantly suppressed IMC growth by approximately 50%. Additionally, the CoSn_3_ phase exhibited a dense, layered structure for Zn contents below 4.8 wt.%. In contrast, the CoSn_3_ in the Co-8.8Zn reaction displayed a duplex structure with two distinct microstructures. In the solid-state reactions at 160 °C, the suppression effect of Zn was more pronounced. The addition of 0.55 wt.% Zn significantly reduced the IMC growth. As the Zn content increased, the suppression effect became more evident. The Co-8.8Zn sample maintained a thin IMC layer, showing over 85% suppression, even after long-term aging. These findings demonstrate that Zn plays a key role in modifying interfacial reactivity and microstructure evolution. Co-Zn coatings with higher Zn content effectively suppress excessive IMC growth, which is crucial for enhancing the thermal and mechanical reliability of solder joints. This makes them promising candidates for surface finishes in microelectronic packaging and advanced lead-free soldering applications.

## Figures and Tables

**Table 1:** Composition of the electroplating Co solution (per liter of solution).

Component	Concentration (g/L or mL/L)	Concentration (M)
Cobalt sulfamate solution (Co(SO_3_NH_2_)_2_, Co > 180 g/L)	500 mL/L	1.527 M
Cobalt chloride (CoCl_2_)	10 g/L	0.077 M
Boric acid (H_3_BO_3_)	40 g/L	0.647 M

**Table 2:** Formulation of the electroplated Co-Zn alloy coatings and results of EPMA compositional analysis.

Adding Amount of ZnSO_4_·7H_2_O (Based on 200 mL of Co Electroplating Solution)	ZnSO_4_·7H_2_O Concentration (M)	Zn Content in Co-Zn Coatings (wt.%)	Average Zn Content and Notation
0.1 g	0.00174 M	0.51–0.62	Co-0.55 wt.%Zn
0.5 g	0.0087 M	1.93–3.14	Co-2.5 wt.%Zn
1.0 g	0.0174 M	4.02–5.62	Co-4.8 wt.%Zn
2.0 g	0.035 M	8.49–9.08	Co-8.8 wt.%Zn

**Table 3:** Coefficients of thermal expansion (CTE) of relevant materials [35]. The CTE of Co-Zn alloys is not reported in literature. Based on the rule of mixtures and the known CTE values of pure Co and Zn, the CTE of Co-Zn alloys with Zn content below 10 wt.% is estimated.

Material	CTE (×10^−6^ K^−1^)
Co	~13
Cu	~16.5
Sn (β-Sn)	~22
Zn	30.2
Co-Zn alloys (estimated)	~14–16 (depending on Zn content)

## Data Availability

The original contributions presented in this study are included in the article/Supplementary Material. Further inquiries can be directed to the corresponding author.

## Supplementary Materials

The following supporting information can be downloaded at: https://www.mdpi.com/article/10.3390/ma18122680/s1, Figure S1: BEI micrographs of the Sn/Co-2.5Zn reaction at 250 °C for (a) 10 min and (b) 30 min; Figure S2: BEI micrographs of the Sn/Co-4.8Zn reaction at 250 °C for (a) 10 min and (b) 30 min; Figure S3: BEI micrographs of the Sn/Co-8.8Zn reaction at 250 °C for (a) 10 sec, (b) 10 min, (c) 30 min, and (b) 30 min; Figure S4: Cross-sectional micrograph of the Sn/Co–2.5Zn/Cu sample reacted at 250 °C for 2 h (identical to Figure 7c in the main text), provided here for reference and further analysis; Table S1: EPMA analysis in Sn/Co-2.5Zn/Cu reaction at 250 °C for 2 h. The analyzed positions were shown in the figure below.
