# Supplementary material for: Interfacial Behavior During Reactions Between Sn and Electroplated Co–Zn Alloys
Source: Materials (Basel). 2025 Jun 6;18(12):2680. doi: 10.3390/ma18122680 (PMC12194586; doi:10.3390/ma18122680)
Supplement: Supplementary file 1 [file materials-18-02680-s001.zip › materials-3666148-supplementary.pdf]

materials

Supplementary material

Interfacial Behavior During Reactions Between Sn and Electroplated Co–Zn Alloys

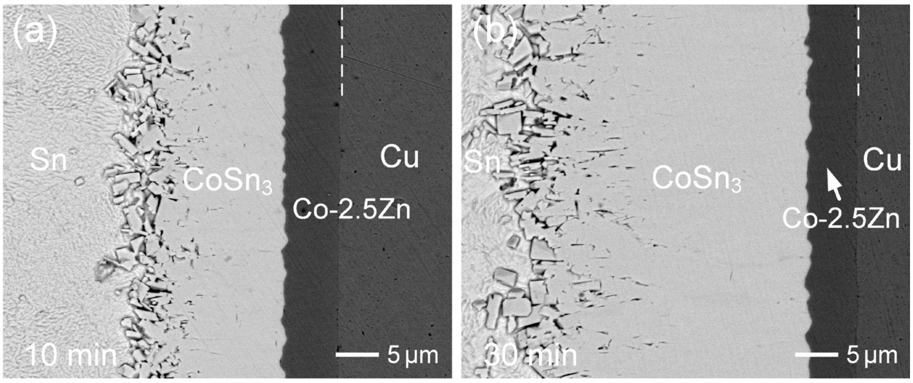

Figure S1. BEI micrographs of the Sn/Co-2.5Zn reaction at 250°C for (a) 10 min and (b) 30 min.

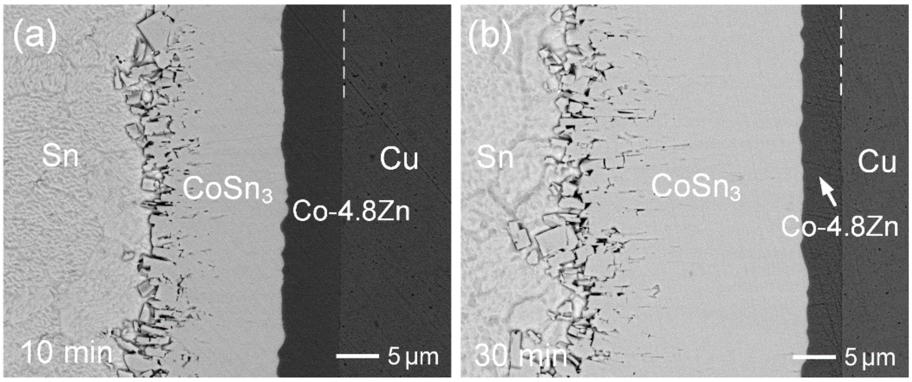

Figure S2. BEI micrographs of the Sn/Co-4.8Zn reaction at 250°C for (a) 10 min and (b) 30 min.

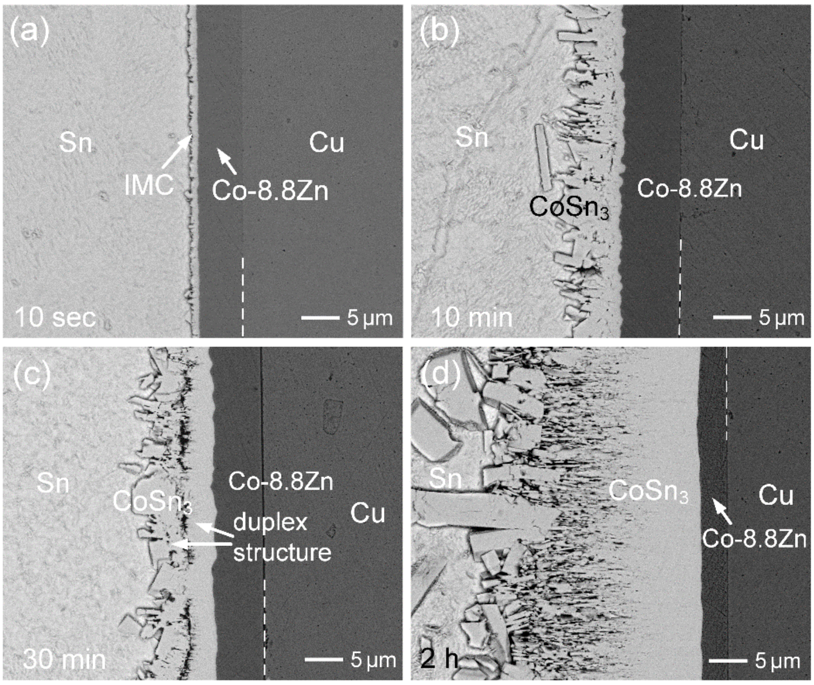

Figure S3. BEI micrographs of the Sn/Co-8.8Zn reaction at 250°C for (a) 10 sec, (b) 10 min, (c) 30 min, and (b) 30 min.

In Figure S3(c), a seam resembling peeling was observed at the interface between the Co-8.8Zn deposit and the Cu substrate. However, this feature is considered a superficial surface artifact rather than a true interfacial separation. Among approximately a dozen specimens with 8.8 wt.%Zn, only one or two exhibited such a seam after undergoing a series of processes, including sample cutting, solder reaction, mechanical polishing, etching, and ultrasonic cleaning. The observed seam was shallow; if it had been a true delamination crack, capillary action would likely have caused water to become trapped within the gap, which would manifest during optical microscopy or SEM examination as moisture release or residue marks. Since no such indications were observed, the seam is presumed to be a minor surface feature. It is speculated that Co-Zn deposits with relatively high Zn content are more susceptible to attack by the Sn etchant solution or mechanical damage during polishing, leading to the formation of this artifact.

Table S1. EPMA analysis in Sn/Co-2.5Zn/Cu reaction at 250°C for 2 h. The analyzed positions were shown in the figure below.

Position	Sn (at%)	Co	Cu	Zn	phases
a	75.75	23.63	0.51	0.11	$CoSn_3$
b	48.34	9.64	41.89	0.13	$(Cu,Co)_6Sn_5$
c	48.42	2.80	48.77	0.01	Cu_6Sn_5

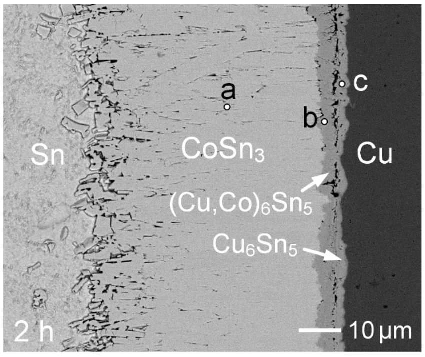

Figure S4. Cross-sectional micrograph of the Sn/Co–2.5Zn/Cu sample reacted at 250°C for 2 h (identical to Figure 7(c) in the main text), provided here for reference and further analysis.
